# Telemedicine in the United States: An Introduction for Students and Residents

**DOI:** 10.2196/20839

**Published:** 2020-11-24

**Authors:** Maryam A Hyder, Junaid Razzak

**Affiliations:** 1 Barnard College New York, NY United States; 2 Department of Emergency Medicine Johns Hopkins School of Medicine Baltimore, MD United States

**Keywords:** telemedicine, telehealth, eHealth, biomedical technology, mHealth, mobile health, COVID-19

## Abstract

Telemedicine refers to the delivery of medical care and provision of general health services from a distance. Telemedicine has been practiced for decades with increasing evidence proving its potential for enhanced quality of care for patients, reduction in hospital readmissions, and increase in savings for both patients and providers. The COVID-19 pandemic has resulted in a significant increase in the reliance on telemedicine and telehealth for provision of health care services. Developments in telemedicine should be structured as complements to current health care procedures, not with the goal of completely digitizing the entire health care system, but rather to use the power of technology to enhance areas that may not be working at their full potential. At the same time, it is also clear that further research is needed on the effectiveness of telemedicine in terms of both financial and patient benefits. We discuss the current and rapidly increasing knowledge about the use of telemedicine in the United States, and identify the gaps in knowledge and opportunities for further research. Beginning with telemedicine’s origins in the United States to its widespread use during the COVID-19 pandemic, we highlight recent developments in legislation, accessibility, and acceptance of telemedicine.

## Introduction

The World Health Organization (WHO) defines digital health as the use of digital technologies for health purposes, a category that encompasses the increasing use of technologies for health services [1]. Related to digital health is telemedicine; telemedicine seeks to harness the growing role of technology to create more effective health care, and is part of a larger movement of digital health services. Table 1 [1,2] provides definitions of various terms used under the broad scope of digital health and their relationship to each other. These terms have some minor differences, but are important to recognize as relevant to the overall discussion of digital health. Our focus in this paper, however, is specifically telemedicine: its history, its uses, and its importance.

**Table 1:** Definitions of digital health terms.

Terms	Definitions^a^
Digital health	The use of digital technologies for health. Encompasses eHealth, mobile health (mHealth), and the use of computer science (such as big data and artificial intelligence).
eHealth	The use of information and communication technologies for health.
mHealth	The use of mobile wireless technologies (such as cell phones) for health.
Telemedicine^b^	The use of information and communication technologies to improve patient outcomes by increasing access to care and medical information.
Telehealth	The most basic engagement of eHealth, involving telecommunications and virtual technology to deliver health care outside of traditional facilities.

^a^According to the World Health Organization [1].

^b^The American Telemedicine Association considers telemedicine to be synonymous with telehealth [2].

In the United States, 76% of hospitals connect with patients using some form of telemedicine [3]. Kane and Gillis identified physicians practicing in radiology (39.5%), psychiatry (27.8%), and cardiology (24.1%) as the most frequent users of telemedicine [3].Telemedicine not only is effective for provider-patient interactions but also creates a more connected network between health care professionals. The specialties reported to most commonly use telemedicine to communicate with other health care professionals were emergency medicine (38.8%), pathology (30.4%), and radiology (25.5%) [3]. With increasing need for a multidisciplinary approach to care and patient-provider partnerships, telemedicine has helped further strengthen connections between patients, health care providers, and other stakeholders. In the face of the COVID-19 pandemic, the relevance of telemedicine has become even more acute. Use of telemedicine has been rapidly promoted, and laws on its coverage are rapidly changing.

Here we provide a background of the increasing use of telemedicine in the United States and emphasize its relevance for medical students and residents. Beginning with telemedicine’s origins in the United States to its widespread use during the COVID-19 pandemic, we highlight developments in legislation, accessibility, and acceptance of telemedicine. Omitted from this review are comprehensive descriptions of the technical aspects of telemedicine, as well as newer developments in technology such as artificial intelligence. This review is meant to be a starting point for students, and thus we omit more technical information for the sake of brevity and focus.

## History of Telemedicine in the United States

In the United States, one of the early uses of telemedicine was established by the National Aeronautics and Space Association (NASA) in 1960, for monitoring astronauts in flight by physicians and medical teams during their mission Project Mercury [4]. NASA designated “medical monitors” to become well-versed in the astronauts’ medical history, while conducting research on the effect of the environment of outer space on the human body. Teams of medical observers were positioned at 18 sites across North America, Europe, Africa, and Australia. Their role was to observe and preserve the health of the astronauts by providing medical advice when needed and consistently evaluating their condition [4]. As NASA demonstrated, using telecommunications to establish contact between health care providers and patients can allow for greater availability of, and access to, health care beyond what was previously conceived as possible. Understanding this great potential that telemedicine holds for increased connectivity, the US National Library of Medicine, in 1966, designated US $42 million for multiple telemedicine projects spanning over 19 years targeted to medically isolated—rural, inner city, and suburban—areas [5].

## Approaches to Telemedicine

### Telemedicine Use in Various Fields of Medicine

In the United States there are several successful and active models of telemedicine and telehealth, demonstrating their potential. In 2013, there were approximately 4 neurologists per 100,000 people in the country, caring for over 700,000 strokes per year [6]. To deal with the shortage of neurologists, several hospitals and offices implemented telemedicine measures specifically for stroke treatment, or telestroke. Telestroke refers to a common method used in emergency departments to access specialist neurologists, reducing the need for in-house experts. Using telemedicine, neurologists can communicate remotely with emergency physicians and patients with stroke and recommend treatment faster than previously available [6]. This is especially helpful for smaller, rural hospitals that do not have specific specialists for a vast range of conditions compared with larger urban medical centers [7]. Hospitals’ use of telestroke enables patients to go to their nearest hospital while still receiving the specialized emergency care they require, and thus all patients are given equal chances of survival [6].

Telemedicine has also been particularly useful in radiology, since nearly all radiology examinations produce digital content, known as teleradiology. In 2014, teleradiology was reported to account for more than half of all telemedicine services performed in the United States [8]. Images and reports collected from either in-person or telemedicine examinations can be transmitted to a remote radiologist, whose report can be sent to the patient’s physician or other health care providers. Particularly in areas where there is a shortage of radiologists, teleradiology supplements that shortage by eliminating the in-person meeting between patient and radiologist, as well as providing quicker readings and results to patients [9].

Even fields that traditionally have depended on face-to-face communications, such as psychiatry, have been able to provide care of comparable quality using telemedicine. As of 2016, a mere 43.1% of the 44.7 million Americans with any sort of mental illness had used mental health services in the United States [10]. Being able to provide care to more Americans with mental illness through telepsychiatry could have a profound effect. The University of Rochester in Rochester, NY, USA created a telepsychiatry program that is available from 8:30 AM to 5:00 PM, Monday through Friday, and is performing about 2000 telepsychiatry consultations per year [10]. A provider simply sends a request for a visit with a patient by computer, and the query is answered by a specialist at the university. The interface of communication is extremely simple, consisting of an iPad on a rolling stand for providers and a video camera for the patient. Nurses are also able to use the interface to record videos of patients in agitated states and securely send the videos to specialists, who can use a patient’s video and medical record to recommend treatment and calming techniques [10].

### Telemedicine for Patients at Home

The implementation of certain at-home monitoring telemedicine and telehealth systems has also been successful. For example, Columbia University in New York, NY, USA created a home telemedicine unit through its Informatics for Diabetes Education and Telemedicine initiative [11]. This home telemedicine unit is capable of videoconferencing, medical data acquisition, sharing of collected data with physicians, web-based access to clinical data, and web access tailored to diabetes education. Data are transmitted through the existing telephone lines of each household, meaning no extra technological installment was needed. Each home telemedicine unit includes glucose and blood pressure monitors, alerts patients if any of their recorded values are abnormal, and indicates whether they should contact a physician [11]. Long-term outcome data are awaited to determine its full success.

At-home telehealth monitoring, especially for patients with chronic diseases, has proven to be beneficial for not only patients but also hospitals. The Patient Protection and Affordable Care Act requires the US Centers for Medicare & Medicaid Services to penalize readmissions to hospitals that occur within 30 days after discharge [12]. This provision incentivizes hospitals to find another way to communicate with and treat patients who may not require readmission, but simply medical attention. A study conducted by Partners HealthCare in 2014 [13] provided in-home monitoring to over 3000 patients with congestive heart failure. Their personal data—blood pressure, weight, heart rate, pulse—were recorded and uploaded daily to a monitoring system that used decision support software to alert patients and nurses of which patients needed attention. The data were transmitted securely through a telephone service to the internet. Over 6 years, hospital readmissions dropped by 44% for these patients, and the program generated savings of over US $10 million. This program led to a more efficient use of health human resources, as it allowed 3 to 4 nurses to care for 250 patients rather than the 4 to 6 patients they would be assigned to in a traditional hospital setting [13]. This example demonstrates the potential of telehealth to reduce health care costs, as well as unnecessary hospital admissions for patients, while also benefitting hospitals through reduced readmission penalties. In addition, this study highlights that telemedicine does not benefit one part of the system over another, but ideally works to create overall improvement in the health care system.

Similar results were seen in the implementation of a home health program for veterans, which used telemonitoring to help manage veterans with chronic illnesses, such as diabetes and depression. The Care Coordination/Home Telehealth program yielded high satisfaction from patients and produced a 19% reduction in the number of hospital visits as compared with their usual care [14]. The program generated savings of nearly US $2000 per patient, and even facilitated independent living of 36% of patients, who otherwise would have qualified for long-term residential care [14]. Through this increased access to knowledge, the goal of health care could be shifted from intervention to prevention, as increased education can promote healthier self-management.

### Telemedicine as an Emergency Tool During the COVID-19 Pandemic

As COVID-19 continues to spread, many hospitals and physician practices have transitioned to telemedicine to conduct nonessential appointments [15]. A study of trends at NYU Langone Health (New York, NY, USA) showed an 80% decline in in-person visits and a 683% increase in telemedicine visits between March 2 and April 14, 2020 [15]. Most routine consultations could be conducted over the phone with both the patient and the physician at home, which is particularly useful for quarantined physicians. Telemedicine can also play an instrumental role in treating patients in hospitals. For example, Aurora Health, based in Wisconsin, modified emergency department procedures to allow for remote patient intake, which can accommodate faster testing [16]. As the number of COVID-19–infected patients increases, using computers and tablets for telemedicine can also reduce staff exposure in both ambulances and hospitals. Tablets can be effectively disinfected and allow patients to be safely isolated [16]. Electronic intensive care units (e-ICUs) dramatically increase the number of patients that health care workers can monitor at once, and as the numbers of hospitalized patients increase, e-ICU systems can help clinicians manage the load. Finally, developers Avera Health, based in South Dakota, are working on mobile home health care units for sick patients who are able to return home [16]. At-home systems provide patient evaluation without overwhelming an emergency room, allow physicians to assign sick patients to hospital beds through telemedicine, and even potentially facilitate at-home testing [16]. Great advancements in telemedicine are being made to help cope with this global pandemic and demonstrate how the emergence of telemedicine can shift the preparedness infrastructure of the health care system.

## Health System Issues for Telemedicine

### Reimbursement and the Interest of Health Care Payors and Insurance Providers

The increasing use of telemedicine has led to increasing interest among payors to cover such services. Several insurance companies, and certain government-funded programs, have expanded their policies adjusting for telemedicine services. In 2018, Medicaid in several states broadened the scope of telehealth and telemedicine services for which they reimburse, thus reducing barriers for their use. For example, California approved reimbursement for substance use disorder services delivered through telehealth; Kentucky prohibited the requirement that a physician must be present with the recipient of health care for reimbursement; and Colorado expanded reimbursement to teledentistry [17].

In light of the current opioid epidemic in the United States, telemedicine can provide a newer and quicker avenue for crisis intervention, which is being recognized by payor groups. For example, Connecticut had previously prohibited prescription of controlled substances through telemedicine, but as of 2018 the state has made an exception for the prescription of drugs that treat opioid use disorder or substance use disorder [17]. Additionally, outside of government-funded health care, 31 states and the District of Columbia have enacted parity laws, requiring private insurers to reimburse providers for telehealth services [18]. This shows the impact telemedicine is making on the health care system. Furthermore, legislation currently being proposed is working toward making the practical use of telemedicine and telehealth even easier and more accessible for patients nationwide. Insurance reimbursement poses a large determinant in the acceptance of, and attitude toward, telemedicine. Government insurance reimbursement policies, in particular, have been shown to influence telemedicine adoption.

A survey conducted by the American Telemedicine Association in 2014 found that hospitals were not billing for telemedicine services because government payers would not pay for them, and neither would major private payers [19]. Furthermore, administrative rules differed for in-person versus telemedicine care, another barrier to reimbursement, and insurance companies ultimately followed the guidelines of their individual states, further hindering standardization of telemedicine billing. This study concluded that, if government payers change their policies to include reimbursement for telemedicine services, private payers are also more likely to change their policies [19]. Insurance reimbursement incentivizes telemedicine adoption for hospitals, and thus administrative and state-level rules would likely adjust as well. Finally, billing and coding processes for telemedicine services must also be better understood in order to enact change [19].

State governments have the power to decide whether they will cover telemedicine practices; and states are not required to submit an amendment to Medicaid if they decide to reimburse for telemedicine (in the same way as they do for in-person services). As of 2015, Medicaid reimbursed for telemedicine services in 46 of the 50 states [20]. Medicare, however, has created strict guidelines and instances for which it will reimburse telemedicine practices. To be eligible for Medicare reimbursement for a telemedicine service, a case must conform to a set of national guidelines; namely, the receiver of telemedicine service must be in a rural geographical location defined as either a Health Professional Shortage Area or a county outside of a Metropolitan Statistical Area [20]. These areas are defined by the Health Resources & Services Administration and can be found on their Medicare Telehealth Payment Eligibility Analyzer on their website [21]. As of July 2019, a small number of exceptions to this geographical requirement were enacted, including treating end-stage renal disease, stroke, and substance use disorder [22]. Since Medicare, in particular, serves elderly and disabled populations, telemedicine could alleviate the stress of needing to meet with a physician in person, but the lack of reimbursement deprives these populations of the benefits of telemedicine and telehealth.

In response to COVID-19, Medicare has temporarily expanded its services to cover telemedicine services outside of the prior designated sites and platforms through which one could qualify for reimbursement. As of March 6, 2020, Congress waived the Medicare requirements that limited provision of telehealth care to rural areas [23]. These services can be conducted from any hospital, office, or place of residence—a promising change in policy [24]. Finally, certain Medicare plans cover the cost of COVID-19 testing. These temporary expansions of coverage can, hopefully, become permanent and further promote the use of telemedicine.

### Licensing and Use of Telemedicine

In the United States, there is not one standardized license a physician receives that is valid throughout the country, but rather a physician’s medical license is issued only for their state of practice. This poses an issue for interstate telemedicine practices, but in 2014 the Federation of State Medical Boards passed the Interstate Medical Licensure Compact to help facilitate interstate practice [20]. The compact allows a physician to apply through their home state medical board for eligibility of a medical license in another state. If the application is approved by their home state, no further verification is required and, after paying a fee to the requested state, they will be issued a medical license [20]. Such legal changes can allow telemedicine to become a “usual” part of the health system in the future. Given the enormity of the COVID-19 health care crisis, many state governments are reducing the licensing restrictions to ensure more effective use of physicians.

### Technology and Usability

Concerns about the quality and security of medical records have previously hampered the adoption of telemedicine. In a study conducted by Resneck et al in 2016, medical residents posed as patients and submitted internet pictures to direct-to-consumer telemedicine websites and apps treating skin diseases. In terms of security concerns, they found that none of the websites asked for the patients’ ID to prove the photographs were not false [25]. Often clinicians were randomly assigned to patients, depriving patients of any autonomy in their choice of health care provider. In some cases patients were assigned to an international physician who did not have a license to practice medicine for their state. Even though the services diagnosed the conditions accurately based on images only, diagnoses were incorrect and missed major dermatologic conditions when a more detailed medical history was required for conditions such as secondary syphilis and eczema [25]. Maintaining quality is critical while attempting to expand access to care, and similar standards apply to care provided through telemedicine and to in-person care.

Important aspects of telemedicine and telehealth adoption are the ease of use and lack of technical prerequisites on the part of both patients and health care facilities, as well as reliability of new technologies. If telephone calls are not adequate as a means of making telemedicine visits, access to videoconferencing poses an accessibility barrier both for patients and for health care providers. On the patient side, access to the internet, video cameras, and software poses an issue; and for health care systems, installation of certain videoconferencing software may breach privacy governance policies [25]. In dealing with COVID-19, there is need for immediate conversion to telemedicine, and the time needed to develop new telemedicine platforms that mirror health care workflows is limited. Therefore, videoconference appointments must be monitored and controlled accordingly until access to more appropriate telemedicine software is available [26]. Keeping this in mind, COVID-19 testing must be integrated into telemedicine systems, so that after telemedicine appointments such patients are not immediately sent to the emergency room, further overwhelming hospital systems. For example, testing sites outside of the emergency room should be set up to accommodate telemedicine workflow, whether it be in other office spaces, in tents, or at the patient’s home with take-home tests [16].

Finally, despite significant improvement in the reliability and ease of use of technology, there remain a few of limitations, such as some loss of nonverbal communication between physician and patient. This includes, for example, a reassuring touch from a physician or a physician’s ability to smell alcohol on a patient’s breath and address alcohol consumption during the visit. These limitations could compromise the quality of health care in some cases from both the patient’s and physician’s point of view [27].

### Acceptability by Patients and Providers

For telemedicine to ultimately be successful, it must be accepted by both patients and physicians. In 2016, a survey conducted in Austria asked patients and health professionals to rank what they perceived as the highest- and lowest-ranked benefit of and barrier to eHealth and telemedicine. With a demographic of 43.2% working in health professions and 56.8% in non–health professions, the overall highest-ranked benefit of eHealth was location-independent access to health care services, and the lowest-ranked benefit was better financing of health care. The highest-ranked barrier to eHealth was data security, and the lowest-ranked barrier was increase of administrative burden [28]. The highest-ranked benefit of telemedicine specifically was location-independent access to health care services, and the lowest-ranked benefit was better relationships between doctors and patients. For telemedicine, the highest- and lowest-ranked barriers were the same as those for eHealth [28].

This study also showed that acceptance of telemedicine may vary depending on socioeconomic factors. Participants were categorized by education level, gender, health profession or non–health profession, and digital age group. Digital age group was defined as 2 groups: digital natives, those who grew up in a digital world (defined by age ≤35 years); and digital immigrants, those who did not grow up in a digital world, but adopted the culture [28]. Participants were asked to evaluate their opinion on their knowledge of telemedicine, reliability of health information, reasonability of data exchange, consistent monitoring of patients with chronic disease, and consistent monitoring of all patients. Non–health professionals were 40% less likely to report high knowledge on telemedicine, but having a university degree led to 64% higher odds for high knowledge on telemedicine. The study also found that digital age was a determinant in evaluating the reliability of online health information: digital immigrants were 44% more likely than digital natives to find online information reliable [28]. These findings demonstrated that education on eHealth is crucial for widespread acceptance of eHealth among diverse populations. Since several digital natives were less likely to trust online information or telehealth services as reliable, education is needed even for digital users on not only general definitions of telemedicine and telehealth, but also their practical implementation and reliability.

For telemedicine and telehealth to be implemented successfully, patients must be educated about and comfortable with their use. Systems of informed consent are an essential part of this process, allowing patients to change their consent as they learn more about the technology being used and implemented—especially those with unintended but potential consequences. For example, a study implementing a voice-response telephone counseling system affected some participants in an unanticipated manner. Participants in this study were asked to call a telephone line daily and report their diet and exercise behavior, and most participants spent around 5 minutes per week speaking with the system [25]. Over the course of the study, patients began to form a relationship with the voice, referring to it with gender pronouns, or even naming their relationship as a friend, doctor, or mentor. Some reported the voice of the phone-line as condescending, leaving them with feelings of guilt after their call. After the study ended, some participants reported that they “missed the voice” of the system and continued to call the line [27]. Had patients been informed of the possibility that they could form a relationship—even if subconsciously—with this telephone line prior to the study, they would have been able to make an informed choice of whether to participate. This example helps elucidate the need for education about eHealth not only for medical professionals but also for those who will be interacting with it in order to promote acceptability.

## Telemedicine and Telehealth in Special Groups

### Patients With Chronic Illnesses

Telemedicine and telehealth can play a large role in care for those with chronic diseases, if approved by both patient and provider. A study conducted in an amyotrophic lateral sclerosis (ALS) clinic held appointments with patients over live videoconferences in place of their regular visit and measured satisfaction surveys from both the patients and care providers. Patients had an overall high level of satisfaction with videoconferencing checkups. They commented that such specialized-care videoconferencing eliminated the need to drive long distances and allowed appointments to be conducted that would have ordinarily been missed or canceled due to bad weather [29]. A symptom of several chronic diseases, including ALS, is fatigue, and thus one of the most popular remarks about the appointments was that videoconferencing was less taxing for patients than their regular in-person visits. Overall, patients felt they received good and high-quality care comparable with an in-person visit. When asked for negative factors of the videoconference appointment, patients stated that they missed the personal physical gestures of reassurance from physicians, such as hugs, during their visits [29].

### Elderly Patients

For elderly patients, doctor’s appointments often provide an outing; the videoconferencing appointment kept these patients at home, depriving them of an opportunity to mobilize [29]. Suggestions made by the patients included better coordination between clinical care and home care, and thus including a briefing between physician and home caregivers in the videoconference. Additionally, patients commented that the technology and software used may require improvements or replacements, as several patients experienced audio lags during their appointments [29].

### Acceptance by Physicians

Geronimo et al [29] also surveyed physicians treating ALS patients for their perspective on the videoconferencing appointments. The most common remark was that patients seemed more comfortable throughout the visit. Additionally, the videoconferencing appointments increased the physicians’ understanding of their patients’ daily life and the kind of home care they were receiving [29]. The main concern, however, was the lack of physical examination, as well as physical contact for reassurance. This study indicated that, although telemedicine provides a convenient avenue for health care, it cannot completely replace the current system. Both patients and physicians felt comfortable and satisfied after their visits, but certain crucial elements of an in-person visit are irreplaceable, such as physical examinations and gestures. Acceptance of and attitudes toward this form of telemedicine also varied by geographical location. Rural clinicians especially appreciated the monitoring function of e-ICU systems, as it allowed them to be involved in providing care without being physically present [30].

### Acceptance by Nurses

Acceptance depends not only on physicians and patients, but also on the other health care providers interacting with these systems, such as nurses. Health care providers working through various e-ICU systems were surveyed for their general attitudes toward its efficacy and sustainability. The survey found that providers who used the e-ICU more frequently had positive reactions, whereas those who used it less were more likely to have a negative view and to suggest that it imposes a greater burden on nurses [30]. Nurses—who often are responsible for monitoring a large load of patients, as well as coordinating inpatient and outpatients matters—felt the monitoring capabilities of telemedicine helped alleviate some of their stress and to dedicate their time effectively [30]. As this study illustrated, if used effectively, telemedicine can maintain high-quality health care while decentralizing stress on providers.

## Future Directions

Table 2 outlines the key future strategies for improving the availability and quality of telemedicine and telehealth services based on a framework from the WHO [1].

Public policy must be compatible with increased eHealth implementation for telemedicine to be practiced regularly. Despite the exceptions to Medicare’s telehealth reimbursement policy, the current regulations prevent widespread adoption and use of eHealth and set a precedence for other large private insurers. The Creating Opportunities Now for Necessary and Effective Care Technologies (CONNECT) Act was introduced to the US Senate in 2017, but has yet to make progress. This act proposes the removal of the geographical restrictions under Medicaid and, if passed, would expand coverage of telemedicine services [18]. As a result, telemedicine services would be given the legitimacy of in-person visits in terms of Medicaid and may inspire other insurers to change their private policies.

**Table 2:** Summary of key strategies for advancement of telemedicine^a^.

Domains of World Health Organization framework	Key strategies
Leadership and governance	Advocating for interstate medical licensure Promoting a universal reimbursement mechanism for telemedicine in all 50 states for Creating public-private telemedicine leadership groups
Strategy and investment	Directing investments for telemedicine programs Engaging private health insurance companies
Services and applications	Educating patients
Standards and interoperability	Standardizing informed consent Creating a standardized medical licensing system or equivalency system between certain states
Infrastructure	Implementing interoperable systems across health systems Developing patient-friendly technologies for home use
Legislation, policy, and compliance	Removing geographic restrictions for Medicaid reimbursement Enacting state legislation to require private insurers to reimburse for telemedicine services
Workforce	Training a specific telemedicine health workforce across specialties Educating future health professionals in telemedicine

^a^Based on World Health Organization recommendations [1].

State legislation is one of the largest determining factors in telemedicine adoption, and it has been shown that, in states that require private insurers to reimburse for telemedicine, adoption rates have increased [31]. For telemedicine to provide genuine and effective location-independent health care, a standardized licensing system or an equivalency system must also be established in the United States as a whole, or between states. There are several approaches to dealing with interstate licensing other than standardization across the country [20]. State governments can review each other’s board examinations and agree to mutually approve medical licensing between them. This solution gives states autonomy in controlling which physicians are eligible to practice telemedicine within their area, while also creating connections between physicians across the country. If legislation is passed legitimizing the role of telemedicine and telehealth in the American health care system, increased education and research on the area can lead to telemedicine being a prominent daily practice for physicians and patients alike.

## Conclusions

We have reviewed the definitions, challenges, and potential future directions for telemedicine in the United States. It is no longer a question of whether eHealth has a role to play in health care delivery, rather it is a matter of making it happen. As we are learning during this COVID-19 pandemic, telemedicine and telehealth are critical to ensuring public health and are poised to become reliable and acceptable methods of seeking care for many conditions.

## Abbreviations

ALS: amyotrophic lateral sclerosis
CONNECT: Creating Opportunities Now for Necessary and Effective Care Technologies
e-ICU: electronic intensive care unit
NASA: National Aeronautics and Space Association
WHO: World Health Organization
